# Temperature-controlled ultrasonication improves protein extraction from soybean okara by various energy-dependent mechanisms

**DOI:** 10.1016/j.ultsonch.2026.108068

**Published:** 2026-09-20

**Authors:** Ben Van den Wouwer, Kristof Brijs, Christophe M. Courtin, Arno G.B. Wouters, Katleen Raes

**Affiliations:** a Ghent University, Research Unit VEG-i-TEC, Department of Food Technology, Safety and Health, Faculty of Bioscience Engineering, Graaf Karel de Goedelaan 46, 8500 Kortrijk, Belgium; b KU Leuven, Laboratory of Food Chemistry and Biochemistry, Department of Microbial and Molecular Systems, Kasteelpark Arenberg 20, 3001 Leuven, Belgium

**Keywords:** *Glycine max*, Ultrasound, Soy protein, Protein extractability, Aggregation, Microstructure, Protein functionality

## Abstract

The food industry by-product soybean okara is a sustainable source of soy proteins, but its proteins have low extractability, hindering efficient protein isolation. This study investigated how temperature-controlled ultrasound treatments (<30 °C) at varying energy inputs improve okara protein isolation. In three independent okara batches, the extractability of about 50% of the proteins was restricted by non-covalent and covalent aggregation, while the other 50% could not be extracted even in the presence of denaturing and reducing agents, suggesting physical inaccessibility. Ultrasound treatments at pH 9.0 with low applied energy (2,000 J/g fresh weight [FW] okara) enabled extraction of physically accessible aggregated proteins (extraction yield 49%). Increasing the applied energy (10,000–40,000 J/g FW okara) further improved the extraction yield, reaching up to 90%, through additional particle and cell disruption. Therefore, temperature-controlled ultrasonication enhances okara protein extraction via two mechanisms: aggregate dispersion at low applied energy and additional matrix disruption at higher applied energies. After precipitation at pH 4.5, protein isolates produced using ultrasound-assisted processes had purities of 43–47 g/100 g dry weight due to pronounced co-isolation of non-protein constituents like lipids. Using higher ultrasound energy levels during extraction improved the foamability of the produced okara protein isolates, likely due to a less extensively aggregated state of proteins in these isolates. However, overall solubilities and foaming performances remained sub-par, requiring further investigation towards improvement. This study provides new insights into how temperature-controlled ultrasonication facilitates the extraction and functionalization of (aggregated) okara proteins.

## Graphical abstract

**Figure:**
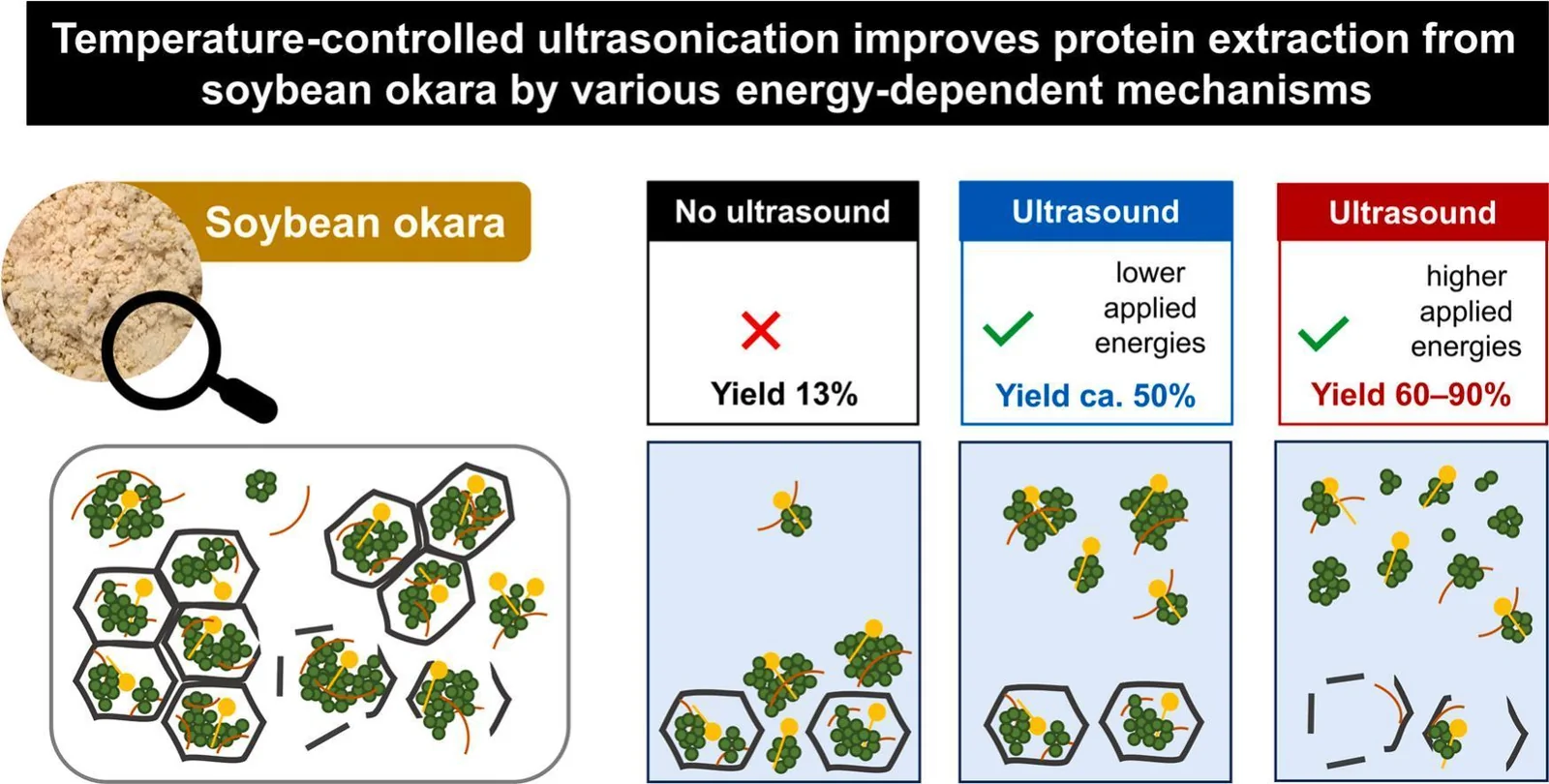

## 1 Introduction

Shifting towards more plant-based protein consumption is considered essential to ensure sustainable food production for the growing world population [1], [2]. Soybeans (*Glycine max* L. Merr) and soybean protein isolates are widely used as ingredients in plant-based alternatives to animal-based products. However, soybean processing generates several by-products, such as okara, the insoluble residue obtained after filtering a soybean-water slurry during the production of soy-based drinks or tofu [3]. Okara is often used in low-value applications due to the lack of cost-effective valorization strategies [4], [5], although its potential as a dietary fiber source has been acknowledged [6], [7], as its fiber content exceeds 50 g/100 g dry weight (DW) [6]. In addition, okara contains 25–30 g/100 g DW proteins [5], [8], [9], [10], but its use as a protein source remains limited. This is mainly due to its complex structure and the alterations in protein properties induced during soybean processing, which make efficient protein extraction challenging.

The okara microstructure primarily consists of cell clusters, ruptured cotyledon cells, seed hull fragments, and aggregated material [8], [11]. As in unprocessed soybeans, the major proteins in okara are 7S globulins (β-conglycinin, trimeric glycoprotein, 180 kDa) and 11S globulins (glycinin, hexamer, 360 kDa) [8], [12], [13]. During soy drink and tofu production, a heat treatment is applied to reduce undesirable flavors, aromas, and anti-nutritional factors [14], [15], [16], but this heating step can be applied before okara removal (Japanese method) or after okara removal (Chinese method) [3]. Therefore, the Japanese method co-produces ‘heated okara’, while the Chinese method co-produces ‘non-heated okara’. Prior studies illustrated that the protein extractability at pH 9.0 and 80 °C from ‘non-heated okara’ is similar to defatted soybean flour (80–85%) [10]. In contrast, the protein extractability from ‘heated okara’ is only 12% at these extraction conditions [17]. This value can be increased up to 48% by extracting at pH 12.6 at 80 °C [17], which is still lower than for ‘non-heated okara’. These findings highlight that okara protein extractability is affected substantially by production process variations, a factor that should be carefully considered when comparing results across studies. These studies further highlight that, particularly for ‘heated okara’, efficient protein extraction is challenging.

High-intensity ultrasound is increasingly acknowledged as an effective strategy to improve plant protein extraction processes [18]. Ultrasound-induced cavitation can facilitate protein extraction through several mechanisms, which can be broadly categorized as effects related to mass transfer, protein properties, and the raw material matrix [18], [19], [20], [21]. Given the complex microstructure of okara and the inherent low extractability of okara proteins, ultrasound may improve protein extraction by enhancing protein diffusion into the extraction solution, by modifying aggregated proteins, and/or by disrupting the okara matrix. These ultrasound-induced effects may occur at different ultrasound intensities or applied energy levels, but this has not been systematically investigated.

The inherent temperature rise during ultrasonication may also influence these effects. For example, distinct diffusion-related enhancements were observed during ultrasound-assisted extraction (0.25 W/mL, pH 9) of sunflower meal proteins at different temperatures (30–50 °C) [22]. Therefore, uncontrolled temperature increases can confound the specific contribution of ultrasound. In one study, ultrasonication of an okara suspension (100 g suspension, 65 W, 0–15 min, pH not specified) increased protein extraction yields from ca. 40% (control sample at 50 °C) to 55–60% [23], but the temperature increased up to 90 °C during ultrasonication. Another study reported that ultrasound-assisted extraction (100 W, pH 12.0, 60 °C, 50 min) improved the protein extraction yield from defatted, milled okara flour up to 80% compared to 36% for a control extraction (pH 12.0, 60 °C, 60 min) [24]. Because extraction temperature strongly influences both yield and protein structural properties [25], distinguishing the effects of ultrasound waves from confounding thermal effects is critical. A temperature-controlled ultrasonication setup enables this distinction, ensuring that observed outcomes can be attributed directly to ultrasound rather than uncontrolled heating [21]. To the best of our knowledge, the mechanisms by which ultrasound affects okara protein extraction at controlled, low-temperature conditions (20–30 °C) has not been systematically investigated.

A comprehensive assessment is required to understand the potential and limitations of ultrasound for improving okara protein isolation, taking into account ultrasound-induced effects on the okara matrix, protein structure, and protein functionality [18]. Foaming properties were the primary functionality of interest considering the challenges associated with using okara proteins in foam-based food applications [17] like plant-based meringues, mousses, cakes, and barista-type applications of plant-based drinks [26]. In addition to potentially increasing protein extractability, ultrasound-assisted isolation may induce structural changes in the okara proteins that enhance their effectiveness as foaming agents.

This study aimed to understand how ultrasound improves soybean okara protein isolation processes. First, protein extractability and aggregation were investigated in three independent batches of ‘heated okara’ by evaluating protein recoveries and molecular weight (MW) distributions in solutions containing different denaturing and/or reducing agents. Next, okara proteins were isolated by extraction (pH 9.0) followed by isoelectric precipitation (pH 4.5), with and without temperature-controlled (<30 °C) ultrasonication during the extraction step. The effects of different applied ultrasound energies on (i) protein yields, (ii) extraction residue structure, and (iii) physicochemical and foaming properties of isolated proteins were comprehensively evaluated.

## 2 Materials and methods

### 2.1 Materials and chemicals

Soybean okara was freshly procured from a soy drink production line at De Hobbit NV (Maldegem, Belgium) in July (batch A), early September (batch B), and mid-September (batch C) of 2022. Okara was the residue after soaking, milling, cooking, and filtration of a soybean-water mixture, thus corresponding to ‘heated okara’. The three okara batches were stored at −40 °C and were used as triplicates to include variation induced by the production process.

Glycerol, Kjeldahl tablets, sodium dodecyl sulfate, sodium dihydrogen phosphate dihydrate, sodium hydroxide, trichloroacetic acid (TCA), and urea were from VWR International (Leuven, Belgium). Acetic acid, aprotinin from bovine lung, bovine serum albumin, bromophenol blue, citric acid, Coomassie Brilliant Blue R250, dithiothreitol, hydrochloric acid, methanol, pepsin from porcine gastric mucosa, phosphorylase b, sodium phosphate dibasic dodecahydrate, soybean trypsin inhibitor, and Tris buffer were from Sigma-Aldrich (Hoeilaart, Belgium). SeeBlue™ Plus2 Pre-stained Protein Standard was from Thermo Fisher Scientific (Waltham, MA, USA).

### 2.2 Determination of okara proximate composition

The okara dry matter content was determined using the AOAC oven drying method 934.01 [27]. Lipid contents of freeze-dried okara were determined by chloroform/methanol/water extraction [28]. Four sequential lipid extractions were performed and the lipid content was determined gravimetrically after combining and evaporating the chloroform phases.

The okara crude protein content (N x 5.7) [29] was determined using the Kjeldahl method (ISO 937:2023) using a SpeedDigester K-436 and K-350 distillation unit from BÜCHI (BÜCHI Labortechnik GmbH, Branch Office Benelux, Breda, the Netherlands). True protein contents were determined similarly, after prior protein precipitation using a 20.0 w/v% TCA solution [30].

### 2.3 Assessment of okara protein extractability and aggregation state

The extractability and aggregation state of okara proteins were assessed following the approach of Van den Wouwer et al. [31]. Okara proteins were extracted using five different solutions containing denaturing and reducing agents, followed by determination of the protein recovery in each solution (Section 2.3.1). The resulting extracts were analyzed by size-exclusion high performance liquid chromatography (SE-HPLC, Section 2.3.2) and gel electrophoresis (Section 2.3.3) to assess differences in protein composition and aggregation state. Analyses were performed in triplicate for each okara batch.

#### 2.3.1 Protein recoveries in various extraction solutions

Proteins were extracted in (i) sodium phosphate buffer (NaP, 0.05 M, pH 6.8), (ii) NaP buffer containing 2.0% (w/v) sodium dodecyl sulfate (NaP+SDS), (iii) NaP+SDS containing 2.0 M urea (NaP+SDS+U), (iv) NaP+SDS containing 1.0% (w/v) dithiothreitol (NaP+SDS+DTT), and NaP+SDS+DTT containing 2.0 M urea (NaP+SDS+U+DTT). Okara was freeze-dried to enable homogenous sampling, and samples were accurately weighed (1.0 mg crude protein scale), after which 1.0 mL of extraction solution was added. Suspensions were vortexed, shaken (60 min, 150 rpm, room temperature), centrifuged (10,000 *g*, 10 min), and filtered (0.45 µm). Samples containing DTT were prepared under nitrogen atmosphere. Before filtration (0.45 µm), an aliquot of the supernatant of SDS-containing samples was taken for separation using SDS-PAGE (Section 2.3.3).

The pellet obtained after centrifugation was freeze-dried, weighed, and its crude protein content (N x 5.7) was determined using an automated Dumas system (Elemental Analyzer model EA1108, Carlo Erba, Hindley Green, UK), following an adapted AOAC method 990.03 [27]. Pellets from extraction with NaP+SDS+U and NaP+SDS+U+DTT were excluded from protein content determination because urea contributes nitrogen to the measurement. The crude protein yield in a given extraction solution was calculated as follows (all on dry basis):

$$Crude\;protein\;yield\;\left(\%\right)=100*\frac{{\mathrm{mass}}_{\mathrm{okara}}*\mathrm{crude}\;\mathrm{protein}\;{\mathrm{content}}_{\mathrm{okara}}-{\mathrm{mass}}_{\mathrm{pellet}}*\mathrm{crude}\;\mathrm{protein}\;{\mathrm{content}}_{\mathrm{pellet}}}{{\mathrm{mass}}_{\mathrm{okara}}*\mathrm{crude}\;\mathrm{protein}\;{\mathrm{content}}_{\mathrm{okara}}}$$

#### 2.3.2 Size-exclusion HPLC analysis

Protein MW distributions in filtered extracts (Section 2.3.1) were determined by separation on a BioSep SEC-S3000 column (Phenomenex, Torrance, CA, USA) using a Shimadzu (Kyoto, Japan) Prominence modular system and detected at 214 nm, using 0.5 mL/min flow rate and 20 µL injection volume [31]. Samples were eluted with NaP (samples extracted in NaP) or NaP containing 0.1% (*w/v*) SDS (samples extracted in NaP+SDS, NaP+SDS+U, NaP+SDS+DTT, and NaP+SDS+U+DTT). Marker proteins were phosphorylase b (97 kDa), bovine serum albumin (66 kDa), pepsin from porcine gastric mucosa (35 kDa), soybean trypsin inhibitor (20 kDa), and aprotinin from bovine lung (6.5 kDa). Markers were prepared and analyzed in the same way as the okara samples.

#### 2.3.3 Protein gel electrophoresis

Unfiltered extracts prepared in SDS-containing solutions (Section 2.3.1) were analyzed by SDS-PAGE [31]. Four parts of the extracts were mixed with one part of sample loading buffer [12.5 mM 2-amino-2-hydroxymethyl-propane-1,3-diol (Tris), 4.0% (w/v) SDS, 30% (v/v) glycerol, 0.004% (w/v) bromophenol blue]. Samples containing DTT were prepared under nitrogen atmosphere using a sample loading buffer containing 1.0% (w/v) DTT. Samples (20 µL) were loaded on a 4–20% (w/v) Mini-Protean TGX Precast polyacrylamide gel (Bio-Rad, Hercules, USA) and analyzed using a Mini-Protean system (Bio-Rad, Hercules, USA). Gels were stained with 0.025% (w/v) Coomassie Brilliant Blue R250 in 40.0% (v/v) methanol containing 7.0% (v/v) acetic acid and de-stained with 5.0% (v/v) methanol and 7.0% (v/v) acetic acid in water.

### 2.4 Control protein isolation

Fresh okara (5.0 g) was weighed and deionized water was added in a 1 to 10 (w/v) ratio. The pH was adjusted to 9.0 using 2.0 M NaOH. Samples were horizontally shaken (150 rpm, 120 min, room temperature) and centrifuged (4,000 *g*, 10 min). The supernatants and residues were separated and an aliquot of the supernatant was weighed, freeze-dried, and weighed again to determine the dry matter yield. Additionally, supernatant aliquots were taken for crude protein content determination using the Kjeldahl method as described in Section 2.2.

After extraction, the remaining supernatant was weighed, and proteins were precipitated by adjusting the pH to 4.5 with 0.1% (w/v) citric acid, followed by incubation at room temperature for 60 min. After centrifugation (4,000 *g*, 10 min), the pellets were redispersed in deionized water and neutralized using 2.0 M NaOH. The resulting samples were freeze-dried, weighed, and subsequently referred to as protein isolates, irrespective of their protein content. The crude protein contents of the isolates were determined using the Kjeldahl method (Section 2.2).

### 2.5 Temperature-controlled ultrasound-assisted protein isolation

Ultrasound-assisted protein isolations were conducted by applying ultrasound during the protein extraction step at pH 9.0 [21]. Briefly, 5.0 g of fresh okara was weighed in borosilicate glass beakers (Duran®; 100 mL, 71 mm height, 51 mm outer diameter), 50.0 mL deionized water was added and the pH adjusted to 9.0, similar to the control process (Section 2.4, 55 g total suspension mass). Samples were treated at 26 kHz using a Hielscher UP200St ultrasound device equipped with a 14 mm diameter probe. A theoretical power level of 100 W was used, corresponding to a theoretical power density of 2 W/mL. The probe was submerged to the middle of the suspension volume. Ultrasound was applied at a 50% duty cycle, consisting of alternating on/off intervals of 0.5 s. Treatments were standardized based on the actual applied energy recorded by the device software. Ultrasonication was automatically terminated once a cumulative energy of 10,000, 50,000, 100,000, or 200,000 J had been reached. The average applied ultrasound power during the treatments can be calculated using the total treatment durations (Fig. 1) and was 30–31 W. The applied energy values corresponded to applied energy levels of 2000, 10,000, 20,000, or 40,000 J/g fresh weight (FW) okara, respectively, which are the units used throughout the manuscript to refer to the different ultrasound treatments. For literature comparison, these energy levels can alternatively be expressed as 200, 1000, 2000, and 4000 J/mL extraction medium (50 mL water) or 182, 999, 1818, and 3636 J/g total suspension mass (55 g, combined mass of okara and water).

**Fig. 1:**
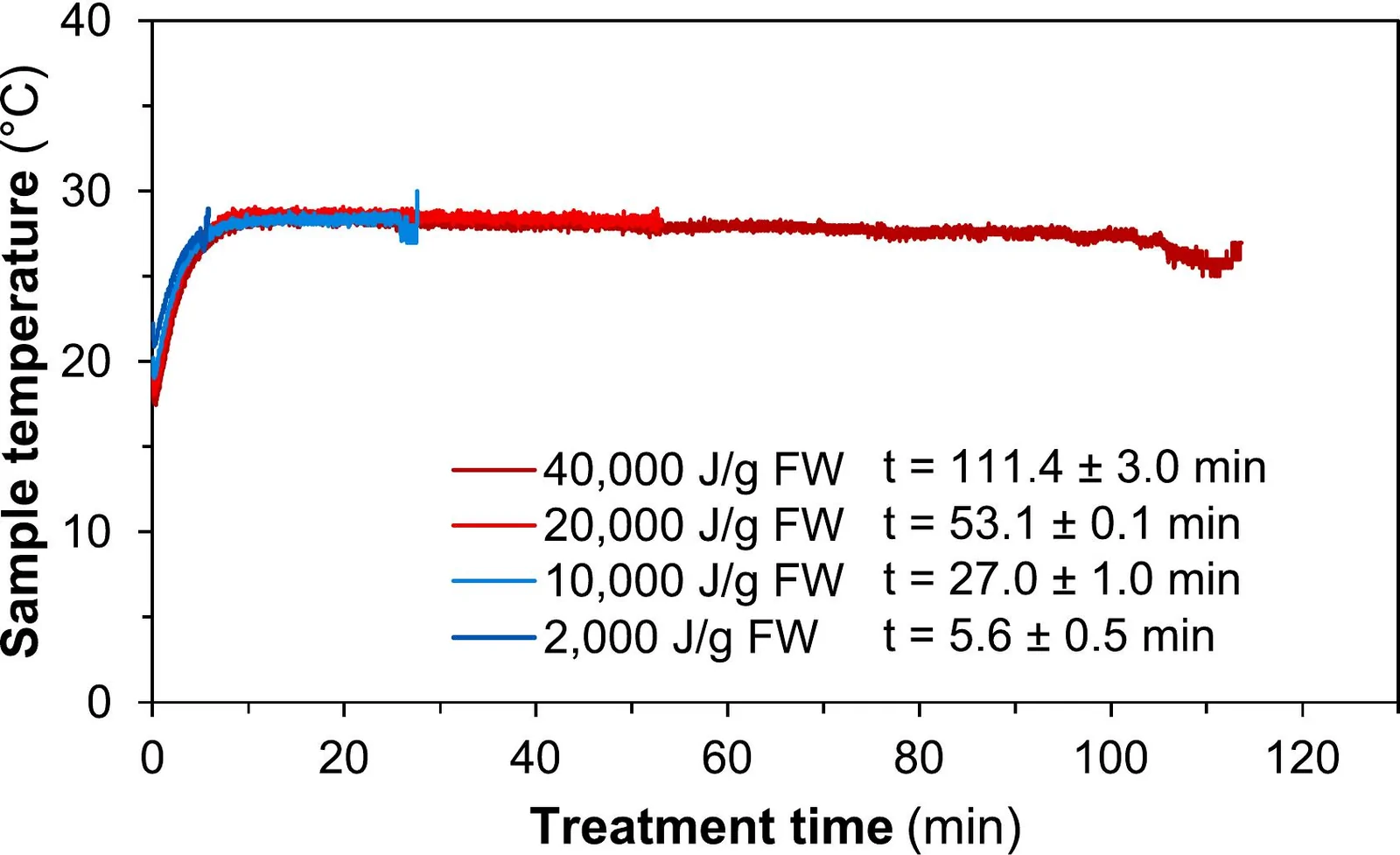
Sample temperature as a function of treatment time for different applied ultrasound energy levels. Average treatment times (t) required to reach the specified energy inputs are indicated. Temperature profiles and treatment times are averages of biological triplicates. FW = fresh weight.

During ultrasonication, samples were cooled in an ice-water bath contained within a Styrofoam box. Temperature was monitored using a built-in temperature probe (Fig. 1). By combining a 50% duty cycle and ice-water cooling, sample temperatures did not exceed 30 °C, irrespective of treatment duration, ensuring that sample temperature did not deviate pronouncedly from room temperature. The ultrasound treatments were varied by altering the applied ultrasound energy, which corresponded directly to different treatment durations (Fig. 1). The most energy-intensive ultrasound treatment (111 min, Fig. 1) was shorter than the control extraction under continuous shaken agitation (120 min, Section 2.4).

After treatment, samples were centrifuged to separate extraction supernatants and residues, and aliquots were collected following the control isolation procedure (Section 2.4). Proteins were precipitated from the extraction supernatants at pH 4.5 to prepare protein isolates (Section 2.4). Lipid contents of isolates produced using the 40,000 J/g FW ultrasound treatment were determined as described in Section 2.2.

### 2.6 Particle size and microscopic analysis of extraction residues

Particle size distributions of residues (pellets) from the extraction step of the control and ultrasound-assisted isolations were determined by dynamic particle imaging with a Sympatec GmbH QICPIC particle size and shape analyzer (Clausthal-Zellerfeld, Germany) using the OASISWET dispersing system with a cuvette gap width of 2.0 mm combined with the M5 lens. Particle sizes were estimated based on a circle of equal projection area, yielding volume-estimated particle size distributions and d_50_ values. The extraction residue microstructure was assessed using light microscopy by sample suspension in deionized water and analysis with a Nikon Eclipse 80i microscope (Nikon Inc., New York, USA). Representative pictures were taken at 40 × magnification.

### 2.7 SE-HPLC analysis of protein isolates and determination of protein aggregation state

Differences in apparent MW distribution and protein aggregation state in protein isolates were assessed using SE-HPLC in a similar way as described in Section 2.3.2. Suspensions of protein isolates were prepared in NaP buffer, NaP+SDS, and NaP+SDS+DTT by weighing on 1.0 mg crude protein basis and adding 1.0 mL solution. Suspensions were vortexed, shaken (60 min, 150 rpm, room temperature), centrifuged (10,000 *g*, 10 min), filtered (0.45 µm), and analyzed using SE-HPLC as described in Section 2.3.2.

### 2.8 Solubility and foaming properties of protein isolates

Solubilities were determined by suspending protein isolates at 6 mg/mL crude protein concentration in 10 mM sodium phosphate buffer (pH 7.0), shaking (150 rpm, 60 min), and centrifuging (10,000 *g*, 10 min) at room temperature. The pellet after centrifugation was freeze-dried, weighed, and its crude protein content determined using the Dumas method (Section 2.3.1). Crude protein solubility was determined as 100% minus the ratio of crude proteins in the pellet to crude proteins in the original sample. Dry matter solubility was determined as 100% minus the ratio of dry matter in the pellet to dry matter in the original sample.

Foaming properties were determined with a standardized stirring test using protein isolate suspensions (50 mL) at 6 mg/mL crude protein concentration [32]. Suspensions were prepared in 10 mM sodium phosphate buffer (pH 7.0) and stirred using a magnetic stirrer (20 min, room temperature). Samples were transferred to graduated glass cylinders (60.0 mm inner diameter) and equilibrated for 10 min in a water bath at 25 °C. Samples were then stirred at 2000 rpm for 70 s with a rotating propeller (45.0 mm outer diameter, 0.4 mm thickness). Using the graduations on the glass cylinders, foam and liquid volumes were recorded at 2, 5, 10, 15, 20, 30, 45, and 60 min after the start of stirring. The foaming capacity and foam stability were determined as follows:

Foamingcapacity(mL)=Foamvolume2minafterthestartofstirring

$$\mathrm{Foam}\;\mathrm{stability}\;(\%)=100\times \frac{Foam\;volume\;60\;\min\;after\;the\;start\;of\;stirring}{Foam\;volume\;2\;\min\;after\;the\;start\;of\;stirring}$$

### 2.9 Statistical analyses

Proximate composition (Section 2.2) and protein extractabilities using SE-HPLC (Section 2.3) were determined for each of the three independent okara batches (Section 2.1) using technical triplicates. For all other analyses (2.4, 2.5, 2.6, 2.7, 2.8), batch-to-batch variation was taken into account by using biological triplicates, and values are reported as means and standard deviations calculated from these biological triplicates. JMP Pro 17 (JMP Statistical Discovery LLC) was used for statistical analyses. Means were compared using one-way analysis of variance and groups were compared using Tukey’s honest differences test (α = 0.05).

## 3 Results and discussion

### 3.1 Characterization of three independent okara batches

#### 3.1.1 Okara composition

Table 1 presents the composition of the three independent batches of ‘heated okara’ used in this study. The dry matter, crude protein, and lipid contents correspond well with literature values [8], [33], [34]. Since the crude and true protein values were similar between batches, all nitrogen in okara was insoluble in 20 w/v% TCA solution and therefore assumed to be of protein origin [35]. These three batches of okara were previously reported to contain similar amounts of fiber (49.4 ± 1.1 g/100 g DW) and pectin (5.3 ± 1.2 g/100 g DW) [36].

**Table 1:** Composition of the soybean okara batches used in the study. Means and standard deviations correspond to technical replicates (n = at least 3). FW, fresh weight; DW, dry weight.

	**Batch A**	**Batch B**	**Batch C**
**Dry matter** (g/100 g FW)	14.4 ± 0.4	17.1 ± 0.3	16.7 ± 0.1
**Crude protein** (g/100 g DW)	23.2 ± 0.9	24.0 ± 2.9	28.0 ± 1.1
**True protein** (g/100 g DW)	23.6 ± 1.6	24.0 ± 0.3	28.4 ± 1.3
**Lipid content** (g/100 g DW)	14.8 ± 0.5	15.3 ± 1.4	14.9 ± 0.5

#### 3.1.2 Okara protein extractability and aggregation state

Protein recoveries in five different solutions were compared, i.e. NaP, NaP+SDS, NaP+SDS+DTT, NaP+SDS+U, and NaP+SDS+U+DTT. This approach allows assessing how protein extractability is affected by various factors such as aggregation and/or physical inaccessibility [31]. The presence of SDS in the extraction solution breaks non-covalent bonds (hydrogen bonds, hydrophobic, and electrostatic interactions), while some non-covalent interactions that are not disrupted by SDS may be disrupted by adding urea [37], [38]. Adding the reducing agent DTT to the extraction solution disrupts disulfide (SS) bonds. Comparing protein recoveries in these different solutions therefore elucidates how different denaturing/reducing agents affect protein extractability. Moreover, the amount of proteins that are inaccessible for the denaturing/reducing agents can be estimated. Studying the apparent MW distributions of the extracts with SE-HPLC or SDS-PAGE allows identification of the extractable protein fractions.

The three okara batches had similar protein recoveries in the same extraction solution (Table 2). The amount of protein extracted by NaP buffer was very limited (0.8–3.1%). Disrupting non-covalent interactions allowed extraction of 13% of the crude proteins (NaP+SDS, Table 2), while additionally disrupting SS bonds allowed extraction of 53% of the proteins (NaP+SDS+DTT, Table 2). Therefore, the extractability of ca. 50% of the proteins in heated okara seems restricted by different types of aggregation, while the other 50% appear to be physically inaccessible for the denaturing and reducing agents, presumably due to extensive aggregation and/or entrapment in cellular structures. Generally, okara proteins have low extractability in aqueous solutions because water-soluble proteins are removed during the production process [8]. For ‘heated okara’, the heating step applied during this process likely induces aggregation that additionally lowers the protein extractability. For the three batches of ‘heated okara’ investigated here, the extractability of about 50% of the proteins seems restricted by aggregation, while they remain accessible to denaturing and reducing agents. The other 50% of okara proteins appear not to be accessible, indicating either pronounced aggregation or entrapment in cellular structures.

**Table 2:** Crude protein recoveries from three batches of ‘heated okara’ in various extraction solutions with denaturing and reducing agents. Means and standard deviations correspond to analytical triplicates for each batch. In the same column, labelling with different letters indicates significant differences between samples (α = 0.05). NaP = sodium phosphate, SDS = sodium dodecyl sulfate, DTT = dithiothreitol.

	**Crude protein recovery** (%)		
**Batch**	**NaP buffer**	**NaP+SDS**	**NaP+SDS+DTT**
A	3.1 ± 3.4 ^A^	13.4 ± 3.8 ^A^	51.6 ± 1.8 ^A^
B	1.5 ± 2.6 ^A^	12.2 ± 3.4 ^A^	54.5 ± 3.0 ^A^
C	1.3 ± 2.2 ^A^	13.7 ± 4.8 ^A^	54.0 ± 2.4 ^A^
	P = 0.7109	P = 0.8633	P = 0.8561

Proteins recovered from different okara batches had similar MW distributions in the same extraction solution (Fig. 2). Moreover, for the same extraction solution, the protein MW distribution profiles were similar to SDS-PAGE patterns of unfiltered extracts (Fig. 3). In line with the crude protein recoveries (Table 2), the chromatograms of NaP buffer extracts showed no peaks (Fig. 2A), whereas the presence of SDS and SDS+urea enabled protein extraction and thus elution (Fig. 2B and C). When DTT was present (Fig. 2D and E), peak areas increased and peaks could be assigned based on the elution times of marker proteins. Following Verfaillie et al. [39], peaks were identified (Fig. 2D and E) corresponding to aggregates (peak I), lipoxygenase and/or urease (peak II), 7S globulin α’, α, and β subunits (peaks III and IV), 11S globulin B-subunits (peak V), and 11S globulin A-subunits (peak VI). Peak VII likely contains overlapping signals of 11S globulin B-subunits and 2S albumins [39]. The corresponding protein fractions were also identified in the SDS-PAGE patterns (Fig. 3), except for peak II, possibly because lipoxygenase and/or urease abundances were too low for providing recognizable band intensity. As discussed elsewhere [39], other minor protein fractions may also contribute to absorbance in SE-HPLC or band intensity in SDS-PAGE, particularly in peaks/bands III, IV, and V.

**Fig. 2:**
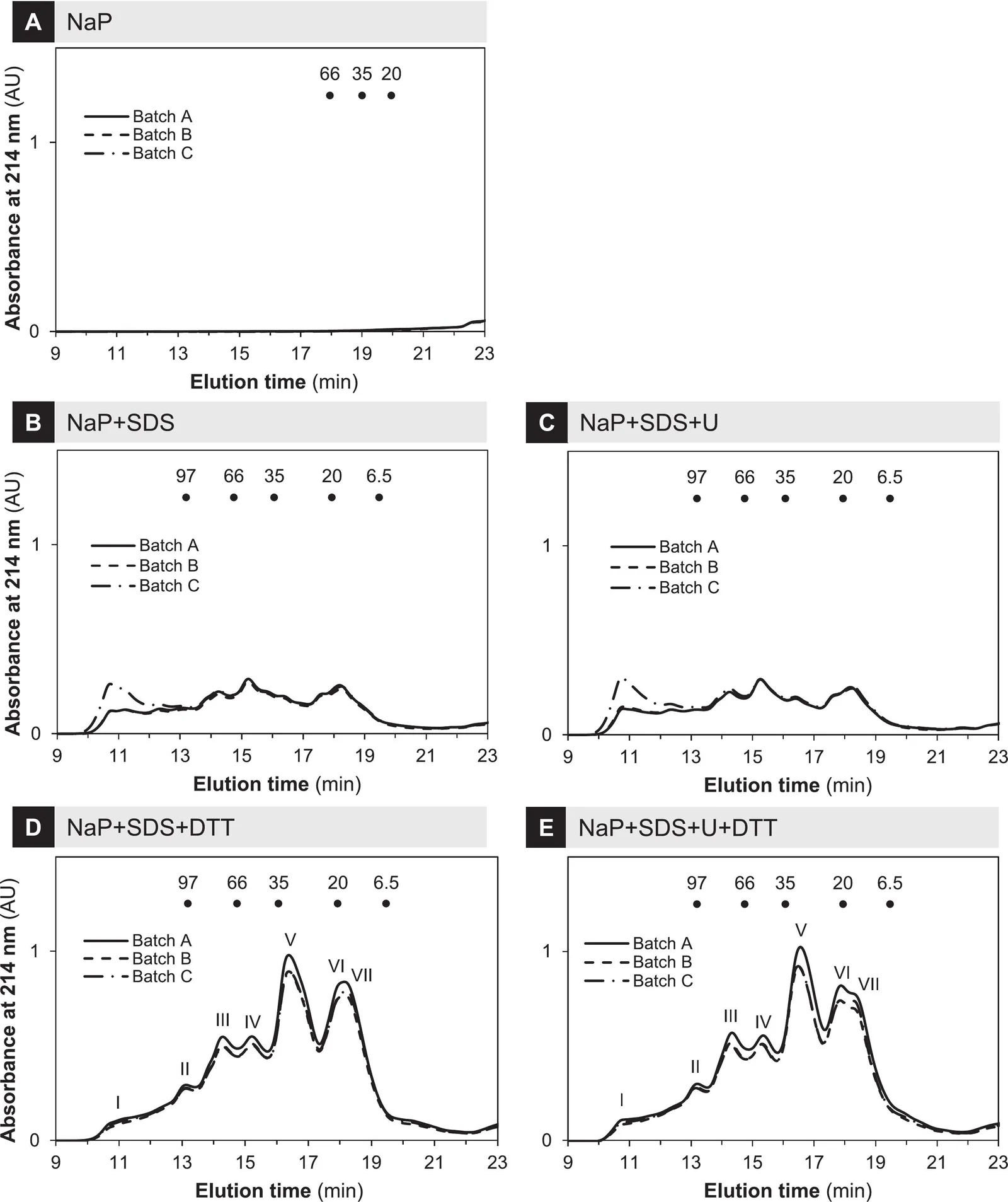
Size-exclusion HPLC profiles of extracts from three batches of ‘heated okara’ (A, B, C) in NaP buffer (A), NaP+SDS (B), NaP+SDS+U (C), NaP+SDS+DTT (D), or NaP+SDS+U+DTT (E). Elution times of marker proteins are given as black dots accompanied by their molecular weight (in kDa). Profiles are averages of analytical triplicates. NaP = sodium phosphate buffer, SDS = sodium dodecyl sulfate, U = urea, DTT = dithiothreitol, AU = arbitrary units.

**Fig. 3:**
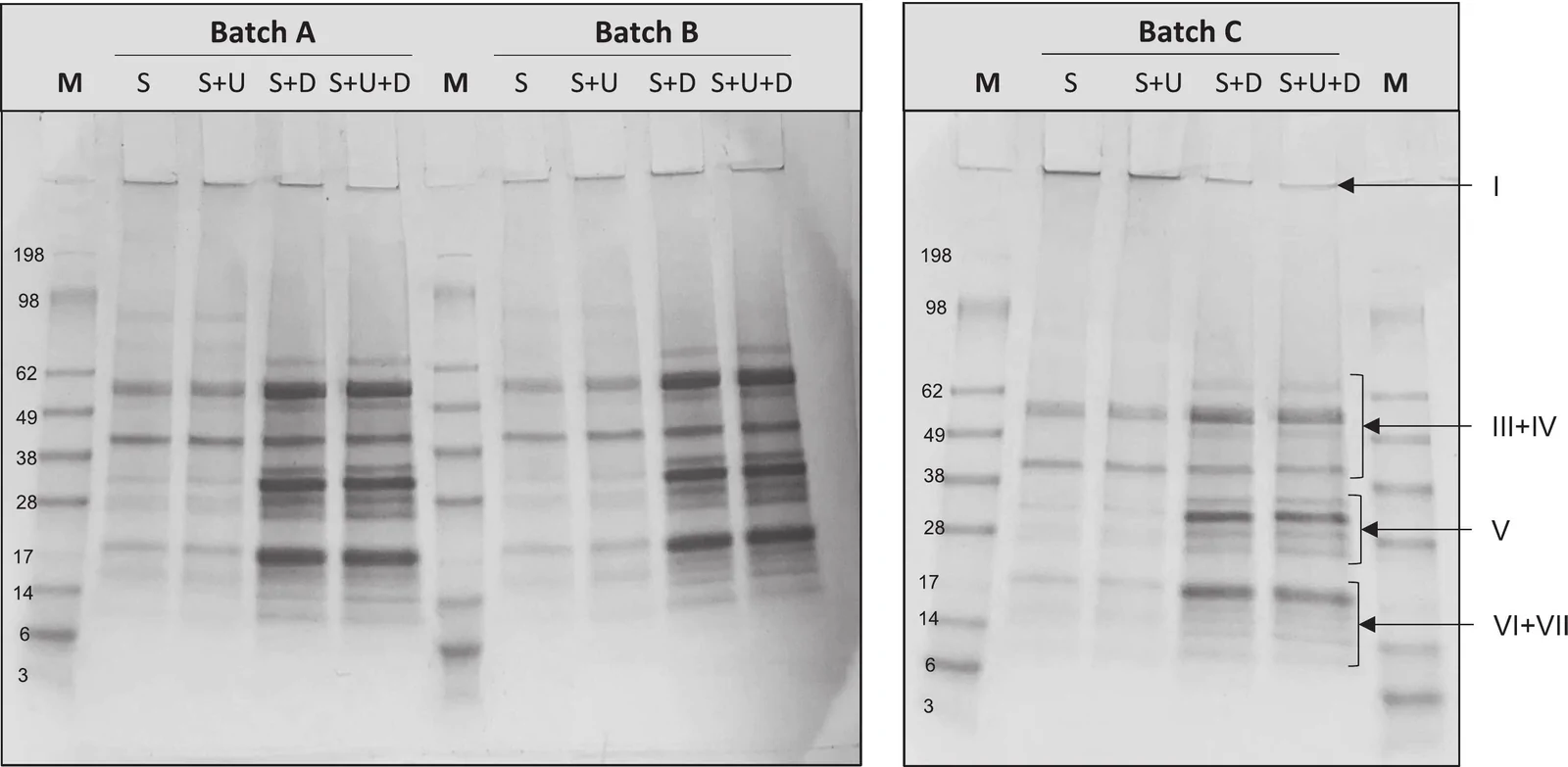
SDS-PAGE patterns of unfiltered extracts from three batches of ‘heated okara’ (A, B, C) in NaP+SDS (S), NaP+SDS+U (S+U), NaP+SDS+DTT (S+D), or NaP+SDS+U+DTT (S+U+D). M = marker, NaP = sodium phosphate buffer, SDS = sodium dodecyl sulfate, U = urea, DTT = dithiothreitol.

The similarity in identified proteins fractions demonstrates that SE-HPLC is a suitable alternative to SDS-PAGE for analyzing protein extractabilities in okara. Furthermore, the inclusion of urea in the extraction solution did not lead to the recovery of additional proteins (Fig. 2C and E, Fig. 3) and did not increase peak areas or band intensities compared to NaP+SDS and NaP+SDS+DTT without added urea (Fig. 2B and D, Fig. 3). Using SDS therefore appears sufficient to estimate non-covalent protein interactions in extractable proteins in ‘heated okara’, although urea may still have aided in the disruption of non-covalent interactions in physically inaccessible, matrix-entrapped proteins. These insights may be relevant for further application of this analytical methodology.

### 3.2 Impact of ultrasound on okara protein isolation

Table 3 gives the protein yields after control and ultrasound-assisted protein isolations. Ultrasonication was conducted during the extraction step at pH 9.0, and protein yields are reported after the extraction and the subsequent precipitation step at pH 4.5. The least energy-intensive ultrasound treatment (5.6 min, 2,000 J/g FW, Fig. 1) extracted 49% of the proteins. Increasing the applied ultrasound energy consecutively increased the protein extraction yield (up to 90%, Table 3). Similar trends were observed for protein yields after the precipitation step of the isolation procedure (Table 3), although some proteins were always lost during the precipitation step. In contrast, the control procedure under continuous shaken agitation for 120 min extracted only 13% of the okara proteins, which further decreased to 2% after the isoelectric precipitation step (Table 3).

**Table 3:** Yields and characteristics of extracts and isolates after control and ultrasound (US)-assisted protein isolations from ‘heated okara’. Means and standard deviations correspond to biological triplicates. In the same column, labelling with different letters points out significant differences between samples (α = 0.05). FW = fresh weight, DW = dry weight.

	**After extraction**			**After precipitation**		
**Isolation method**	**Crude protein yield** (%)	**Dry matter yield** (%)	**Crude protein content** (g/100 g DW)	**Crude protein yield** (%)	**Dry matter yield** (%)	**Crude protein content** (g/100 g DW)
Control	13.2 ± 1.8 ^A^	14.1 ± 2.9 ^A^	23.5 ± 4.0 ^A^	2.3 ± 0.4 ^A^	5.1 ± 1.8 ^A^	35.3 ± 15.4 ^A^
US – 2,000 J/g FW	49.4 ± 11.9 ^AB^	33.7 ± 2.8 ^B^	40.2 ± 3.8 ^B^	41.1 ± 11.2 ^B^	24.4 ± 2.7 ^B^	46.0 ± 1.2 ^A^
US – 10,000 J/g FW	59.7 ± 11.7 ^BC^	41.2 ± 3.3 ^B^	39.8 ± 0.3 ^B^	50.0 ± 9.8 ^B^	30.4 ± 2.0 ^B^	45.2 ± 2.1 ^A^
US – 20,000 J/g FW	77.4 ± 17.1 ^BC^	50.8 ± 3.6 ^C^	42.1 ± 6.1 ^B^	65.5 ± 15.8 ^B^	38.5 ± 3.9 ^C^	46.4 ± 1.0 ^A^
US – 40,000 J/g FW	89.1 ± 18.5 ^C^	56.2 ± 2.6 ^C^	43.5 ± 2.6 ^B^	72.1 ± 17.1 ^B^	44.9 ± 2.9 ^C^	43.8 ± 0.6 ^A^
	P = 0.0004	P < 0.0001	P = 0.0005	P = 0.0003	P < 0.0001	P = 0.3289

This substantial difference in protein isolation efficiency between the least energy-intensive ultrasound treatment and the control indicates that the mechanism driving ultrasound-assisted extraction is not merely diffusion-driven, but rather results from ultrasound-induced alterations in the protein or matrix properties. Moreover, because ultrasonication times were consistently shorter than the extraction time in the control process (Fig. 1), the observed increase in protein extraction yield with increasing applied ultrasound energy suggests that okara protein isolation is enhanced through energy-dependent mechanisms. In food matrices, such effects are mainly attributed to sono-mechanical effects, originating from acoustic cavitation phenomena that result from the growth and collapse of microbubbles in the extraction medium during high-power ultrasonication [20], [40]. In addition, ultrasound may also affect food proteins through radical-mediated sono-chemical effects [41], [42].

Extracts and isolates obtained using ultrasound-assisted processes had similar purities, regardless of the applied ultrasound energy (Table 3), with relatively low purities of 43–47 g/100 g DW for the obtained protein isolates. Meanwhile, applying more ultrasonic energy did increase protein yields (Table 3), resulting in improved protein extraction and isolation compared to the control process. The absence of increased protein contents of extracts and isolates therefore illustrates that applying ultrasound enhanced co-extraction of other constituents from the okara matrix, besides proteins. Subsequently, these co-extracted constituents also seem to co-precipitate with the proteins during the isoelectric precipitation step, eventually resulting in similar purities for isolates obtained using various applied ultrasound energies, regardless of differences in protein yield. In line with this, dry matter yields also drastically increased by applying ultrasound during extraction (Table 3), with treatments of 20,000 J/g FW (53 min, Fig. 1) and 40,000 J/g FW (111 min, Fig. 1) extracting more than 50% of the okara dry matter.

### 3.3 Impact of ultrasound on the particle size and microstructure of extraction residues

To better understand observed yield increases during ultrasound-assisted isolations, particle size distributions and microstructures of the extraction residues were assessed (Fig. 4). Extraction residues from the control process had heterogenous size distributions, with the largest abundance of particles in the size range of 300–1000 µm (Fig. 4A). In general, extraction residues from ultrasound-assisted isolations showed an increasing abundance of 100–300 µm particles as the applied ultrasound energy increased, while the abundance of 300–1000 µm decreased (Fig. 4A). The abundance of particles smaller than 200 µm was highest for the 40,000 J/g FW ultrasound treatment. Since the approximate sizes of soybean cotyledon cells are 15–20 μm by 70–80 μm [11], [43], only the 40,000 J/g FW treatment induced pronounced cell disruption, further evidenced by a higher abundance of the 10–100 µm fraction and lower d_50_ value than for residues from the control and 2,000 and 10,000 J/g FW ultrasound-assisted isolations (Fig. 4A, P = 0.0009). These observations align well with the microscopic analysis (Fig. 4B). Herein, extraction residues from the control process contained large cellular structures, while the matrix was increasingly more disrupted for ultrasound treatments with applied energy of 10,000 J/g FW or higher (Fig. 4B).

**Fig. 4:**
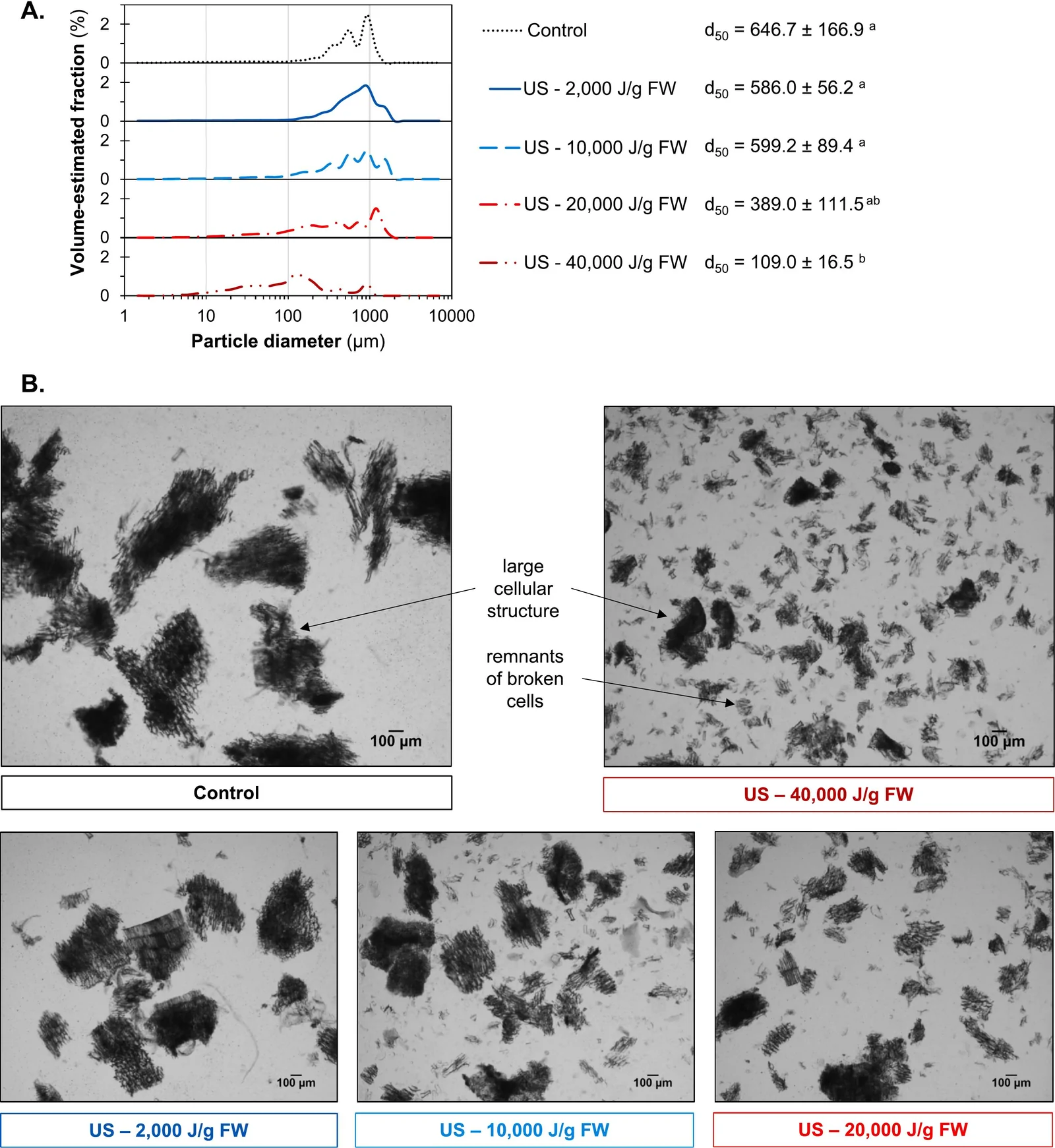
Structural characterization of extraction residues from control and ultrasound (US)-assisted protein isolations from ‘heated okara’. A. Volume-estimated particle size distributions and d_50_ values. Profiles, means and standard deviations correspond to biological triplicates. Values followed by the same letter are not significantly different (α = 0.05). B. Representative micrographs. Scale bars represent 100 µm. FW = fresh weight.

The extraction residue from the 2,000 J/g FW ultrasound-assisted isolation had a particle size distribution (Fig. 4A) and microstructure (Fig. 4B) similar to the residue from the control process. Still, without inducing pronounced matrix disruption effects, this lower-energy ultrasound treatment extracted 49% of the proteins, compared to only 13% for the control process (Table 3). This yield increase at lower applied ultrasound energies therefore seems caused by protein (aggregate) dispersion effects and not by matrix disruption. In line with this, the extraction yield of 49% is similar to the protein recovery in NaP+SDS+DTT obtained from the okara protein extractability assessment (53%, Table 2, Section 3.1). This may indicate that the 2,000 J/g FW ultrasound treatment could effectively disperse protein aggregates that were physically accessible, despite their aggregated state. When the applied ultrasound energy increased, these effects seem to be accompanied by additional matrix disruption effects (Fig. 4), further increasing the protein extraction yield (Table 3).

A reduction in okara particle sizes by ultrasonication (100 g suspension, 65 W, 15 min, 50 °C) has previously been linked to protein extraction yield increases [23]. In that study, effects were attributed to disruption of protein bodies and/or protein aggregates since no pronounced matrix or cell disruption effects were microscopically observed [23]. The discrepancy with the ultrasound-induced matrix disruption observed in the present study seems to originate from differences in the applied ultrasonic energy. Preece et al. applied energy levels up to 168 J/g suspension mass, achieving protein yields up to 60% [23], while the present study applied higher energy levels (182–3636 J/g total suspension mass, Section 2.5), achieving protein yields up to 90% (Table 3). Although another study did report cell disruption after ultrasound treatment of okara [44], sample sizes were not specified, preventing calculation of applied energy levels. Therefore, the present study is the first to illustrate that ultrasonication can improve okara protein extraction via multiple mechanisms that occur at different applied energy levels. Ultrasonication facilitates the extraction of physically accessible aggregated okara proteins at low applied energies (2,000 J/g FW), which is accompanied by okara matrix disruption only at applied energies of 10,000 J/g FW or higher.

### 3.4 Impact of ultrasound on the molecular weight distribution, aggregation state, solubility, and foaming properties of isolated okara proteins

#### 3.4.1 Protein molecular weight distribution and aggregation state

Fig. 5 shows the SE-HPLC analysis of protein isolates in various solutions, allowing to assess their aggregation state. Due to the low isolation yield obtained using the control process (2.3%, Table 3), these isolates were not included in this and subsequent analyses. Analysis of protein isolates in NaP buffer showed no peaks (Fig. 5A), illustrating that isolated okara proteins retained their aggregated state after isolation, irrespective of the applied ultrasound energy. When analyzed in NaP+SDS or NaP+SDS+DTT, peaks can be distinguished. Following the peak identification (Section 3.1.2), the most pronounced differences occurred in the void volume peak, i.e. peak I (Fig. 5C), which corresponds to aggregates. Peak I had the highest intensity for isolates obtained using the 40,000 J/g FW ultrasound-assisted process, and seemed to increase in intensity with increasing applied ultrasound energy (Fig. 5C). The intensity of this void volume peak was even more pronounced when samples were analyzed in NaP+SDS (9–12 min elution time, Fig. 5B), indicating abundant presence of aggregates that cannot be disrupted by SDS. Both for NaP+SDS (Fig. 5B) and NaP+SDS+DTT (Fig. 5C), this void volume peak increased in intensity with increasing applied ultrasound energy, while other peaks showed minimal differences in intensity. Since analyses were conducted on a constant protein basis, this increase in total peak area indicates a greater amount of less extensively aggregated proteins, i.e. proteins with a size low enough to pass the SE-HPLC guard column filter (ca. 30 nm) when analyzed in NaP+SDS and NaP+SDS+DTT. Therefore, this suggests that processes with more energy-intensive ultrasonication produced okara protein isolates containing less extensively aggregated proteins.

**Fig. 5:**
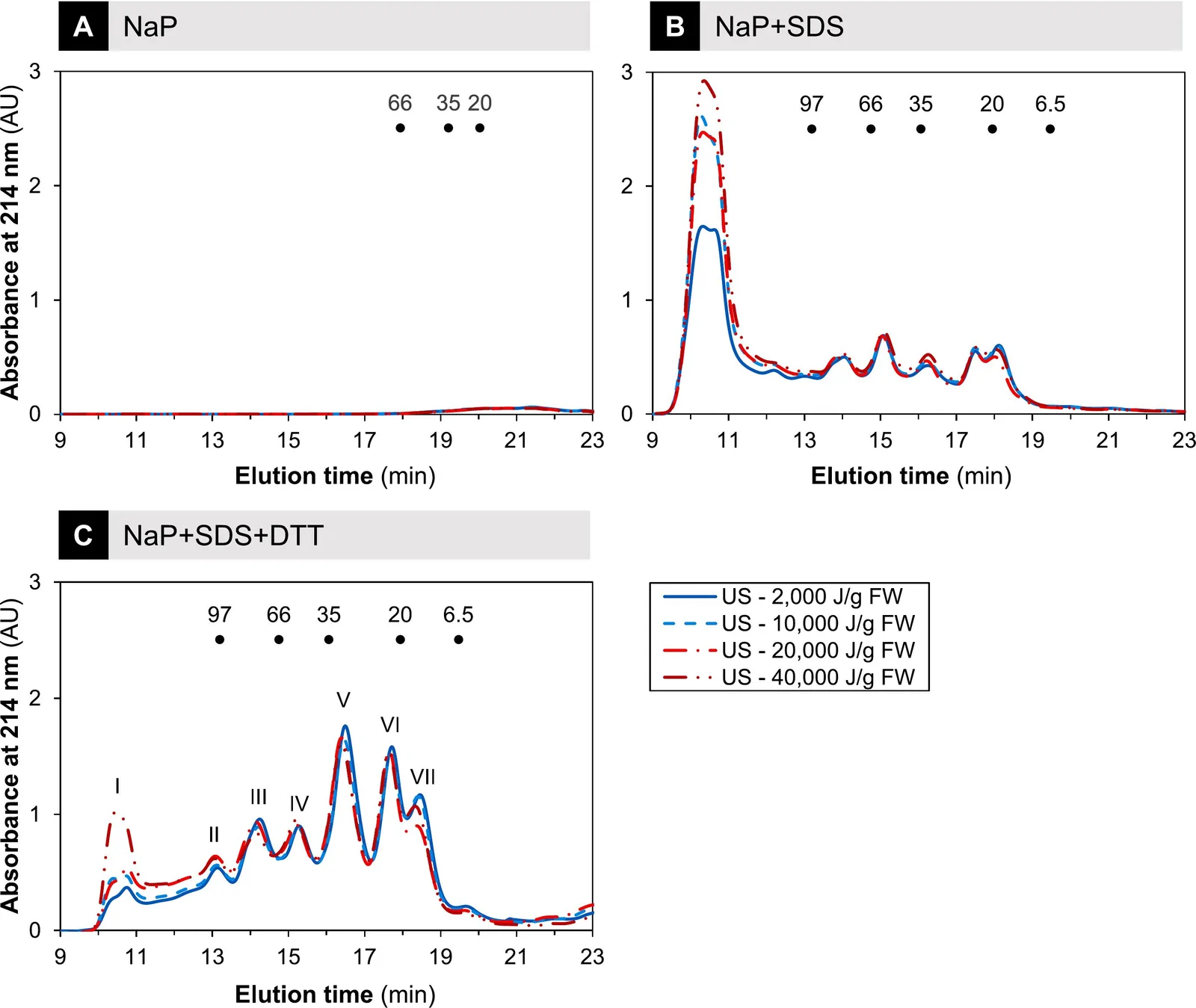
Size-exclusion HPLC profiles of okara protein isolates produced using ultrasound (US)-assisted isolations. Protein isolates were analyzed in NaP, NaP+SDS, and NaP+SDS+DTT. Elution times of marker proteins are given as black dots accompanied by their molecular weight (in kDa). Profiles are averages of biological triplicates. NaP = sodium phosphate buffer, SDS = sodium dodecyl sulfate, DTT = dithiothreitol, AU = arbitrary units.

Since the lower-energy ultrasound treatment did not induce matrix disruption effects (Section 3.3), its higher extraction yield (49%, 2,000 J/g FW) compared to the control (13%, Table 3, Section 3.2) is likely attributable to ultrasound-induced dispersion of aggregated okara proteins. Herein, temperature-controlled ultrasonication likely disrupted aggregates, rendering them extractable, while such effects did not occur during mechanical shaking for 120 min at the same pH. Ultrasound has previously been reported to markedly improve the solubility of aggregate-containing protein isolates, particularly when applied under alkaline conditions [45], similar to pH 9.0 applied during the extraction step in this study. When the applied ultrasound energy was increased, protein extraction was further improved by additionally disrupting the okara matrix (Section 3.3). Additionally, the resulting okara protein isolates also contained less extensively aggregated proteins. This observation suggests that either proteins entrapped inside okara cellular structures were less aggregated than physically accessible proteins, and/or that higher-energy ultrasound treatments disrupted protein aggregates during extraction. Reduced protein aggregate sizes following ultrasonication have previously been reported for okara proteins [24], [46] and aggregated soy proteins [47], and have been linked to changes in intermolecular protein interactions [22], [41], [48]. Moreover, ultrasonication can induce protein unfolding and affect free thiol (SH) groups and disulfide bonds in plant proteins [41], [48]. These structural changes may result from cavitation-related sono-mechanical effects (e.g., shearing or aggregate fragmentation) [42] and/or sono-chemical effects (e.g., free radical-mediated reactions) [41].

#### 3.4.2 Solubility and foaming properties

Fig. 6 shows the foam volume evolution over time for foams produced from isolates obtained via ultrasound-assisted isolations. Table 4 gives the dry matter and crude protein solubility, as well as the determined foaming capacity and foam stability. The amount of foam produced from the okara protein isolates was low, irrespective of the applied ultrasound energy during extraction (Fig. 6). The foams were also rather unstable over time (Fig. 6), with similar foam stabilities between the treatments (Table 4, P = 0.2253). Interestingly, protein isolates obtained using the 40,000 J/g FW ultrasound-assisted process had significantly higher foaming capacity than those obtained with 2,000 J/g FW (P = 0.0309, Table 4). The process involving the 40,000 J/g FW ultrasound treatment also increased the protein yield (Section 3.2, Table 3) and the isolated proteins were less extensively aggregated (Section 3.4.1, Fig. 5). Previous studies have similarly related smaller particle sizes of ultrasound-treated soy proteins to improved foaming capacity, while the foam stability remained unaffected [49]. The results reported here suggest that ultrasonication can be incorporated into protein isolation processes to simultaneously increase the protein yield and modify the protein aggregation state, thereby influencing functionality.

**Fig. 6:**
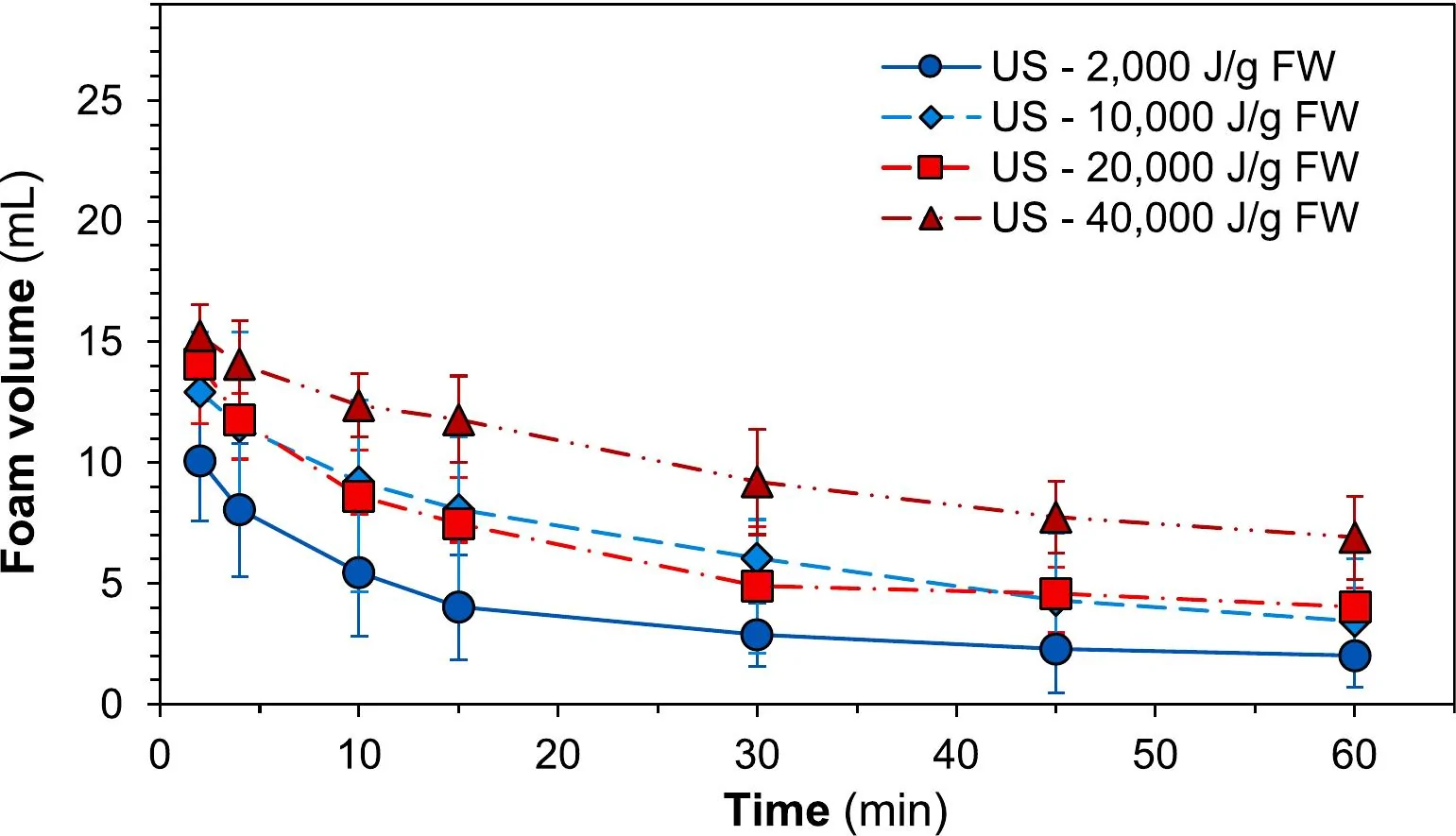
Foam volume over time of stirred suspensions of okara protein isolates produced using ultrasound (US)-assisted isolations. Means and standard deviations correspond to biological triplicates. Lines are solely to improve readability and do not represent real trends. FW = fresh weight.

**Table 4:** Dry matter solubility, crude protein solubility, foaming capacity, and foam stability of okara protein isolates produced using ultrasound (US)-assisted isolations. Means and standard deviations correspond to biological triplicates. In the same column, different letters point out significant differences between samples (α = 0.05). FW = fresh weight.

**US energy input during isolation**	**Dry matter solubility** (%)	**Crude protein solubility** (%)	**Foaming capacity** (mL)	**Foam stability** (%)
US – 2,000 J/g FW	11.4 ± 5.4 ^A^	10.9 ± 5.9 ^A^	10.1 ± 2.5 ^B^	21.1 ± 16.4 ^A^
US – 10,000 J/g FW	6.7 ± 0.9 ^A^	3.2 ± 5.2 ^A^	12.9 ± 1.5 ^AB^	26.5 ± 11.0 ^A^
US – 20,000 J/g FW	12.9 ± 2.0 ^A^	10.8 ± 2.9 ^A^	14.1 ± 1.3 ^AB^	28.6 ± 14.3 ^A^
US – 40,000 J/g FW	11.6 ± 1.7 ^A^	3.5 ± 3.4 ^A^	15.1 ± 1.3 ^A^	45.7 ± 12.6 ^A^
	P = 0.1535	P = 0.1367	P = 0.0309	P = 0.2253

Nevertheless, irrespective of the applied ultrasound energy during extraction, the isolated okara proteins had relatively poor foaming properties, with foaming capacities below 20 mL (<40% of initial suspension volume) and foam stabilities below 50% (Table 4). This is likely related to the low protein isolate solubility, which remained below 15% regardless of the used isolation process (Table 4). This contrasts with reports of protein solubility improvements after ultrasound-assisted isolation from matrices such as sunflower meal [22] and rice bran [50], which contain (mostly) native, non-aggregated proteins. The low solubility observed here suggests that, although ultrasound-assisted extraction allowed effective isolation of aggregated okara proteins (Section 3.2) and influenced the protein aggregation state (Section 3.4.1), the isolated proteins retain an aggregated state, which seems too pronounced to induce solubility improvements in the produced okara protein isolates. The small improvement in foaming capacity for isolates produced at higher applied ultrasound energies therefore does not originate from increased solubility, but likely originates from enhanced foam formation by smaller-sized, insoluble aggregates, e.g. due to ultrasound-induced changes in surface hydrophobicity or molecular flexibility [9].

Notably, ultrasound-assisted processes enhanced co-isolation of other constituents alongside proteins (Section 3.2.1), resulting in isolates with relatively low purities of 43–47 g/100 g DW (Table 3). These values are similar to those reported by Fierens et al. of 45–50 g/100 g DW for okara proteins extracted at pH 12.6 at 80 °C [17]. In the present study, the isolate obtained with the 40,000 J/g FW ultrasound treatment, i.e. the process with the highest protein yield (Section 3.2, Table 3) and the highest level of matrix disruption (Section 3.3, Fig. 4), had a lipid content of 33 ± 4 g/100 g DW. Taking into account the okara lipid content (Table 1), the lipid co-isolation yield is estimated at ca. 45%, indicating simultaneous extraction and precipitation due to protein-lipid interactions. Similar effects have been reported for soybeans, where flour particle size and extraction pH similarly influence protein and lipid extraction, resulting in their co-extraction [43]. Natural protein-lipid interactions exist for some protein types in soybeans [52], while additional interactions may be induced upon heating [53], potentially leading to more pronounced protein-lipid interactions in ‘heated okara’ than in soybean flour. Moreover, ultrasonication may promote interactions between proteins and co-isolated constituents [42], [54], [55]. These induced interactions may influence the techno-functional performance of proteins [55], with the presence of lipids in particular been reported to impact the foaming performance of plant proteins [56], [57]. Herein, apolar lipids can induce anti-foaming effects, rapidly de-stabilizing foams [58], [59]. The potential role of co-isolated lipids in the functionality of okara protein ingredients has not been explicitly studied, but previous studies reported that defatted okara protein isolates produced at 80 °C (protein content 59 g/100 g DW) had foaming properties similar to a commercial soy protein isolate [34], while ultrasound-assisted isolates from defatted okara (protein content not specified) had lower foaming capacity but similar foam stability than commercial soy proteins [51]. Therefore, the poor foaming properties of the okara protein isolates produced in the present study may originate from (combined effects of) a low solubility due to a persisting aggregated state of the proteins and/or (ii) the presence of co-isolated lipids.

Fig. 7 gives a schematic overview of the mechanistic insights gained in this study. Both protein aggregation and protein physical inaccessibility restricted protein extraction from ‘heated soybean okara’, i.e. okara exposed to heat during prior processing (Section 3.1). Temperature-controlled ultrasound treatments at pH 9.0 effectively dispersed aggregated proteins, enhancing their extraction at low applied energy levels (3.2, 3.3). Increasing the applied energy, and consequently the treatment time, further improved protein yields by additional particle and cell disruption within the okara matrix. At higher energy inputs, isolated proteins were less extensively aggregated than those isolated using lower energy levels, which may have contributed to improved foamability (Section 3.4.2). Generally, ultrasound-assisted processes were associated with substantial co-isolation of non-protein constituents, resulting in protein isolates with relatively low purity, limited solubility, and poor foaming properties. While proteins isolated using a process involving higher applied ultrasound energies were less extensively aggregated (Section 3.4.1), this did not result in solubility differences (Table 4). This suggests that ultrasound-assisted extraction allows isolating aggregated okara proteins, but that their aggregated state remains too pronounced to induce solubility improvements, irrespective of the applied ultrasound energy. Interactions with lipids or other co-isolated constituents, as well as additional ultrasound-induced interactions, may even be related to the aggregated state of the isolated okara proteins, which can in turn influence the protein solubility or foaming performance.

**Fig. 7:**
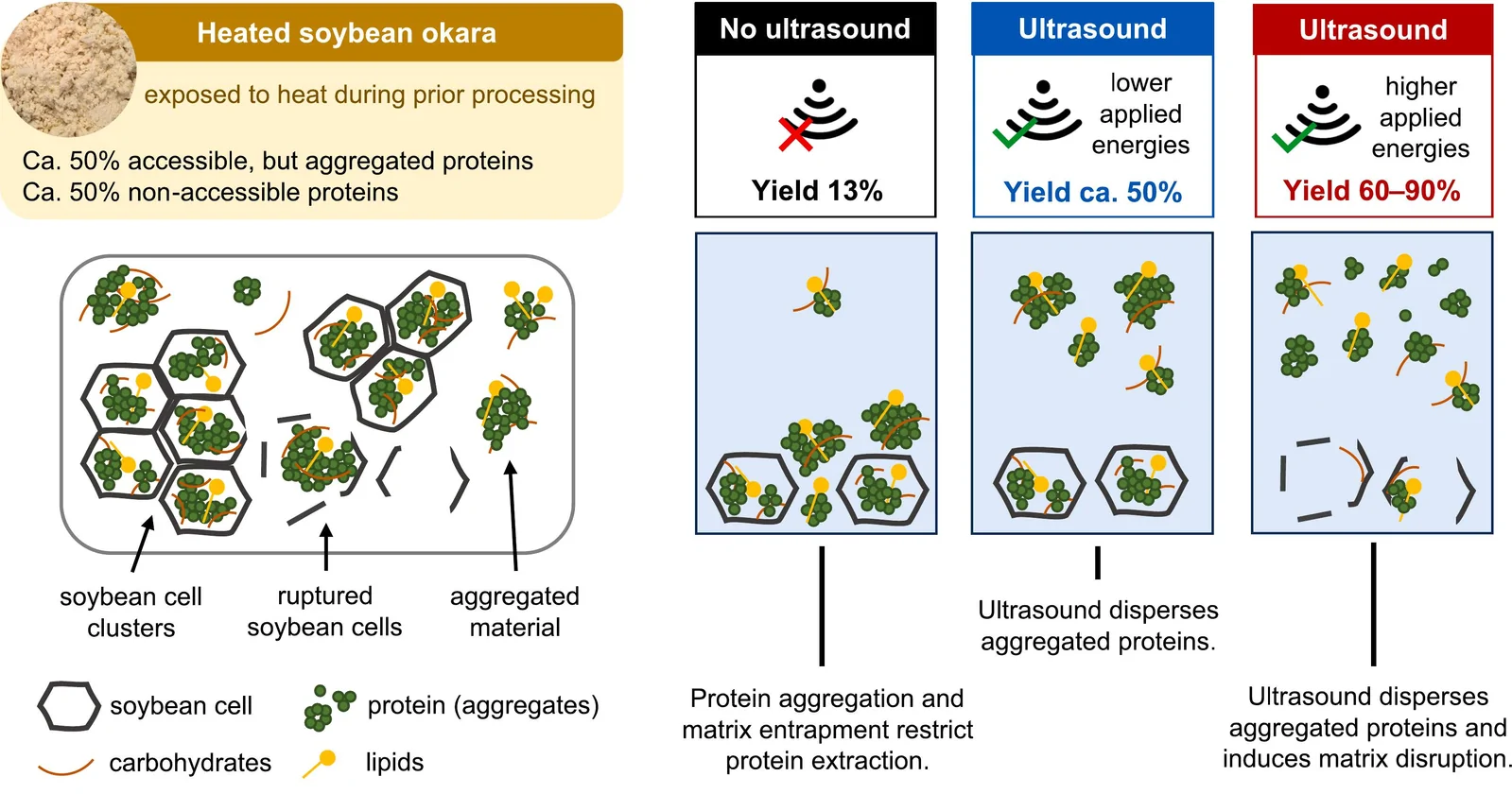
Schematic overview of the energy-dependent mechanisms by which temperature-controlled ultrasound treatment affects protein isolation from ‘heated soybean okara’, i.e. okara exposed to heat during prior processing.

## 4 Conclusions

This study demonstrates that ultrasound can improve protein extraction from okara under controlled temperature conditions, thereby eliminating confounding temperature-related effects. This improvement is driven by multiple mechanisms that depend on the applied ultrasound energy (Fig. 7). Despite this, ultrasound-assisted processes yielded protein isolates with relatively low purity, limited solubility, and poor foaming properties. Purification or modification strategies may be considered to improve foaming performance, or alternative functionalities can be explored. Moreover, further research should aim at unraveling the interplay between ultrasonication and the associated temperature rise, and how this interplay affects protein extractability, structure, and functionality. Furthermore, elucidating how ultrasonication influences interactions between proteins and other matrix components is needed to minimize co-extraction of non-protein constituents during protein isolation.

## CRediT authorship contribution statement

**Ben Van Den Wouwer:** Writing – original draft, Visualization, Validation, Methodology, Investigation, Formal analysis, Data curation, Conceptualization. **Kristof Brijs:** Writing – review & editing, Supervision, Resources, Project administration, Funding acquisition, Conceptualization. **Christophe M. Courtin:** Writing – review & editing, Investigation. **Arno G.B. Wouters:** Writing – review & editing, Investigation, Methodology, Conceptualization. **Katleen Raes:** Writing – review & editing, Supervision, Resources, Project administration, Methodology, Funding acquisition, Conceptualization.

## Declaration of competing interest

The authors declare that they have no known competing financial interests or personal relationships that could have appeared to influence the work reported in this paper.
